# FPGA-Based Real-Time Embedded System for RISS/GPS Integrated Navigation

**DOI:** 10.3390/s120100115

**Published:** 2011-12-22

**Authors:** Walid Farid Abdelfatah, Jacques Georgy, Umar Iqbal, Aboelmagd Noureldin

**Affiliations:** 1 Trusted Positioning Inc., Calgary, AB T2L 2K7, Canada; E-Mail: jgeorgy@trustedpositioning.com; 2 Electrical and Computer Engineering Department, Queen’s University, Kingston, ON K7L 3N6, Canada; E-Mails: umar.iqbal@queensu.ca (U.I.); aboelmagd.noureldin@rmc.ca (A.N.); 3 Electrical and Computer Engineering Department, Royal Military College of Canada, Kingston, ON K7K 7B4, Canada

**Keywords:** embedded systems, FPGA, soft-core, land vehicle navigation, Global Positioning System, inertial sensors, Kalman filter

## Abstract

Navigation algorithms integrating measurements from multi-sensor systems overcome the problems that arise from using GPS navigation systems in standalone mode. Algorithms which integrate the data from 2D low-cost reduced inertial sensor system (RISS), consisting of a gyroscope and an odometer or wheel encoders, along with a GPS receiver via a Kalman filter has proved to be worthy in providing a consistent and more reliable navigation solution compared to standalone GPS receivers. It has been also shown to be beneficial, especially in GPS-denied environments such as urban canyons and tunnels. The main objective of this paper is to narrow the idea-to-implementation gap that follows the algorithm development by realizing a low-cost real-time embedded navigation system capable of computing the data-fused positioning solution. The role of the developed system is to synchronize the measurements from the three sensors, relative to the pulse per second signal generated from the GPS, after which the navigation algorithm is applied to the synchronized measurements to compute the navigation solution in real-time. Employing a customizable soft-core processor on an FPGA in the kernel of the navigation system, provided the flexibility for communicating with the various sensors and the computation capability required by the Kalman filter integration algorithm.

## 1. Introduction

### 1.1. Navigation

Navigation is the science which comprises the methods and technologies to determine the time varying position, velocity and attitude of a moving object by utilizing either sensor-based or satellite-based measurements or by integrating the measurements from both navigation systems [1].

The Global Positioning System (GPS) is a satellite-based, absolute-positioning navigation system, developed by the US Department of Defense (DoD) in the early 1970s, used to provide time, position and velocity information [2]. Although, the navigation solution that is provided by GPS is sufficiently accurate, in the order of meters to centimeters, especially when augmented with other satellite-based or ground-based augmentation systems, it is unable to fulfill the requirements of continuity and reliability in some situations. As a satellite-based navigation system, GPS requires a line-of-sight (LOS) between the receiver’s antenna and the satellites. In urban canyons, tunnels, and other GPS-denied environments, the LOS requirement can’t be always met, resulting in GPS outages caused by GPS signal blockage, interference, jamming and multi-path effects. Signal interruption is one of the main reasons that affects the continuity and reliability of the GPS navigation solution. Due to the mentioned reasons, GPS can’t provide a continuous and reliable solution when used as a stand-alone navigation system, and a better navigation solution can be obtained by integrating the measurements from one or more sensor-based systems, with GPS measurements.

The Inertial Navigation System (INS) is a sensor-based, self-contained, dead-reckoning navigation system in which measurements at a high sample rate, provided by an inertial measurement unit (IMU) are used by a navigation processor to compute the position, velocity and attitude of the moving object relative to a known starting position, velocity and attitude [3]. The IMU consists of a triad of accelerometers and a triad of gyroscopes. Measurements are integrated twice for accelerometers and once for gyroscopes to yield position and attitude. The calculated navigation states (*i.e.*, position, velocity and attitude) drift with time due to the needed integrations and the sensors errors (such as biases, scale factors, and noise) which lead to unbounded accumulation of errors. Therefore, inertial sensors alone are unsuitable for accurate positioning over an extended period of time. The advantages and disadvantages of INS show that its characteristics are complementary with those of absolute positioning systems, such as GPS. Thus, proper integration of the measurements from both INS and GPS can provide great value by mitigating each other problems. The development of Micro-Electro-Mechanical Systems (MEMS) [4] based inertial sensors, made the utilization of MEMS-based INS for consumer applications more affordable as they can be provided at a lower cost. Furthermore they are small in size, light weight, and have low power consumption. However, the errors present in the inertial sensors, materialize by a greater magnitude when using MEMS-based inertial sensors.

Instead of integrating the GPS with a full IMU [5], containing three accelerometers and three gyroscopes, a reduced inertial sensor system (RISS) which utilizes only one gyroscope and an odometer or wheel encoders is integrated with GPS to provide a 2D positioning solution [6,7]. With the assumption that the vehicle stays in the horizontal plane most of the time, the vehicle speed derived from the odometer is used together with the heading information obtained from the gyroscope to determine the velocities along the East and North directions, from which the vehicles’ longitude and latitude are determined. The position, velocity and heading errors are estimated by a Kalman filter (KF) relying on RISS dynamic error model and GPS position and velocity updates [6]. This method requires a dynamic error model for RISS, a measurement model for GPS updates, and a stochastic model of the gyroscope sensor error. Since GPS has a relatively consistent, long-term accuracy, it is used to update both RISS position and velocity components and thus prevent the long-term growth of the RISS errors. On the other hand, the accurate short-term information provided by the RISS is used to overcome GPS outages and multi-path errors. In case a GPS outage occurs, KF operates in prediction mode only, correcting the RISS information based on the system error model. More discussion on the land-based navigation systems, vehicular dead-reckoning and their errors is presented in [8–12].

### 1.2. Embedded Systems

Embedded systems are data processing systems that are embedded into larger systems (such as telecommunication equipment, transportation systems and consumer electronics) and not normally visible to the user [13]. Most embedded devices are designed to perform one dedicated function [14] which requires collecting data from the surrounding environment via a set of sensors, then manipulating the data according to a specific algorithm resulting in useful information for the users. Embedded systems are different than general purpose computer systems in many aspects. These systems differ in that they are required to be dependable, efficient and meet a set of real-time constraints derived from the system to be designed in order not to jeopardize the quality to be provided by the system [13–18]. Another characteristic of embedded systems is its method of software development, known as cross-platform development, where the embedded system’s (*i.e.*, target) software is developed on a host system which employs a general-purpose processor [18]. There exist several hardware platforms that can be used for developing an embedded system such as microcontrollers (μC), digital signal processors (DSP), field programmable gate arrays (FPGA) and application specific integrated circuits (ASIC) [17,19–22]. Choosing one platform over the other depends not only on the requirements of the system to be developed such as the performance, power consumption, cost per chip, but also on the ease of the tools accompanied by a specific platform to assist the developers in producing the system within the constrained system cost and project time.

The ultimate goal, after which a navigation algorithm is developed, is the realization of the algorithm on an embedded system where the measurements from the different sensors are acquired and synchronized, after which the navigation algorithm integrating these time-aligned measurements can be applied to output a real-time solution at a defined rate. The transition from the algorithm research to realization is a crucial step in assessing the practicality and effectiveness of the navigation algorithm and consequently the developed embedded system, either for a proof-of-concept or to be accepted as a product. In the transition process, there is no unique methodology that can be followed to design an embedded system. Depending on the platform of choice, different methodologies are used by the system designers to produce the final product. For example, designers which use processor-based cores such as microcontrollers, or digital signal processors make use of embedded software oriented methodologies to develop their systems, as their main design is revolving around firmware development rather than hardware. However, designers which use FPGAs as their development platforms, have the flexibility to use either the processor-based approach, developing their system entirely in firmware or hardware-based approach, developing their systems entirely in hardware. In other designs in which FPGAs are used, the overall system can be partitioned into hardware and firmware.

The main contribution of this paper is the implementation of an embedded real-time navigation system based on the 2D RISS/GPS navigation algorithm.

## 2. 2D-RISS/GPS Integrated Navigation System

The RISS/GPS algorithm is an integrated navigation algorithm that integrates GPS with self-contained sensors that, unlike GPS, does not suffer from blockages which lead to the interruption of the positioning solution or multipath that leads to the degradation of the positioning solution. GPS helps calibrate the errors in the self-contained sensors that otherwise can cause the positioning error to grow unbounded. As self-contained sensors, the presented navigation algorithm uses a gyroscope whose axis is aligned with the vertical axis of the body of the moving platform and the platform speed readings from either wheel encoders (in case of the mobile robot) or from vehicle odometer (in case of a full sized land vehicle). The benefit of this integrated system for land-based navigation over the traditional INS/GPS integration that utilizes a full IMU (inertial measurement unit) is: (i) the elimination of the two horizontally aligned gyroscopes of the full IMU; and (ii) the use of the vehicle speed to derive velocity directly instead of using accelerometers to derive velocity in the full IMU setting. The first benefit mentioned above is because, during a GPS outage, in a full IMU setting the positioning error is proportional to the cube of the outage duration and the residual uncompensated horizontal gyroscopes bias drifts after the compensation for these biases by the state estimation technique used (such as KF). This is the worst and most influential error in traditional INS/GPS solutions during outages. Taking advantage of the fact that land vehicles move mostly in the horizontal plane and that any off-plane motion is extremely small compared to the in-plane motion, these two gyroscopes and their errors are totally removed from the presented system. The second advantage of the presented system is the use of the vehicle speed readings (from wheel encoders or odometer) together with non-holonomic constraints on land vehicles to derive the velocities instead of relying on accelerometers to derive velocity. Calculating velocity from accelerometers involve a mathematical integration operation, while their calculation form speed readings and heading does not involve such operation. The problem with mathematical integration operations is that it will cause any bias error to lead to a growing unbounded error with time. Using accelerometers, the error in velocity will be proportional to the outage duration and the residual uncompensated accelerometers bias drifts after the compensation for these biases by the state estimation technique used (such as KF); while the position error will be proportional to the square of duration and the uncompensated biases. So the presented RISS/GPS navigation solution will be better than the traditional solution an order of magnitude in duration of the outage because of the elimination of the first integration operation.

### 2.1. RISS Mechanization

RISS Mechanization [6,7] is the process involved in transforming the measurements of a RISS system acquired in the body frame (*i.e.*, vehicle frame) into position, velocity and attitude. It is a recursive process based on the initial conditions, or the previous output and the new measurements.

For a vehicle moving in a 2D plane, five navigation states are computed consisting of two position parameters, two velocity parameters and one orientation parameter. The 2D RISS system readings (**ω_z_** for the gyroscope and **V_forward_** for the odometer) are given in a coordinate frame known as the body frame (b-frame) while the position is expressed in the earth-centered, earth-fixed (ECEF) geodetic coordinate frame and the velocity and attitude are usually expressed in another coordinate frame known as the local-level frame. The local-level frame coordinates are formed from a plane tangent to the Earth’s surface fixed to the current location of the moving object and hence it is sometimes known as a “Local Tangent” or “Local Geodetic” plane.

**Figure 1.**
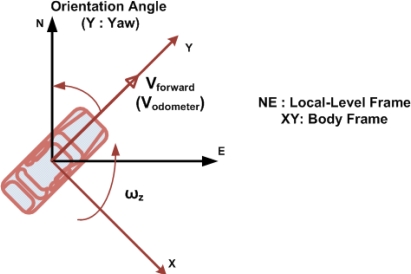
The RISS System Measurements in the b-frame.

In 2D, the position parameters are the latitude (**φ**) and the longitude (**λ**), the velocity parameters are the East (**V_East_**) and North (**V_North_**) velocities, and the only orientation parameter is the azimuth (**A**). Figure 1 shows the measurements of the 2D-RISS system in the body frame, with respect to the local-level frame in which the results are expressed.

The equations that describe the RISS mechanization, which comprises the computation of the vehicle’s position, velocity and orientation, are given below. The rate of change of the yaw angle (where Yaw = −Azimuth) equals the gyroscope measurement, after removing the stationary and non-stationary components, is given by:

(1) $$A_{k+1}=A_{k}-(\omega_{z(k+1)}.T_{s}-\omega^{e}\cdot \text{sin}(\phi_{k}).T_{s}-\frac{V_{\mathrm{east}(k)}\cdot \text{tan}(\phi_{k})}{R+h}.T_{s})$$

where:

*Y* = Yaw angle (radians)
*A* = Azimuth angle (radians)
*ω_z_* = Angular velocity measured by the gyroscope (radians/second)
*ω^e^* =Earth’s angular velocity (radians/second)
*φ* = Vehicle’s Latitude (radians)
*V_East_* = Vehicle’s East component of velocity (meters/second)
*R_N_* = Normal radius of curvature of the Earth’s ellipsoid (meters)
*h* = Vehicle’s altitude (meters).

The subscript (*k*) stands for the previous time epoch; the subscript (*k* + *1*) stands for the current time epoch and *T_s_* stands for the sampling time, the time after which the gyroscope provide a new measurement. Initially, when (*k* = 0), all the variables with the subscript (*k*) on the right hand side of Equation (1), are initialized from the GPS measurements.

The East and North velocities can then be computed using the following two equations:

(2) $$\left(\begin{array}{c} V_{\mathrm{East}(k+1)}\\ V_{\mathrm{North}(k+1)} \end{array}\right)=\left(\begin{array}{c} V_{\mathrm{forward}(k+1)}\cdot \text{sin}(A_{k+1})\\ V_{\mathrm{forward}(k+1)}\cdot \text{cos}(A_{k+1}) \end{array}\right)$$

where:

*V_North_* = Vehicle’s North component of velocity (meters/second)
*V_forward_* = Vehicle’s speed derived from odometer or wheel encoders in the forward direction of the vehicle (meters/second).

After computing the East and North velocities of the vehicle, the position of the vehicle is then computed, specified by the latitude and longitude from the following equations:

(3) $$\left(\begin{array}{c} \phi_{k+1}\\ \lambda_{k+1} \end{array}\right)=\left(\begin{array}{c} \phi_{k}\\ \lambda_{k} \end{array}\right)+\left(\begin{array}{cc} 0 & \frac{1}{R_{M}+h}\\ \frac{1}{\left(R_{N}+h)\cdot \text{cos}(\phi_{k+1}\right)} & 0 \end{array}\right)\cdot \left(\begin{array}{c} V_{\mathrm{East}(k+1)}\\ V_{\mathrm{North}(k+1)} \end{array}\right)\cdot T_{s}$$

where:

*λ* = Vehicle’s Longitude (radians)
*R_M_* = Meridian radius of curvature of the Earth’s ellipsoid (meters).

### 2.2. RISS/GPS Data Fusion Using KF

**Figure 2.**
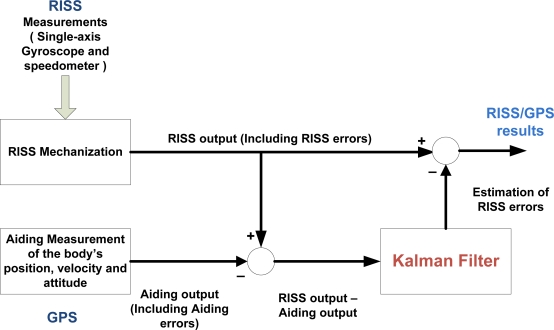
RISS/GPS Integrated Navigation Solution.

The conventional KF method is used, in a closed-loop, loosely coupled fashion, to fuse the position and velocity measurements from the GPS with the RISS computed position and velocity. KF [5,12,23] is a state estimation technique that can integrate measurement data to obtain state estimate by recognizing that the measurements are noisy, and might have only a small effect on the state estimate, or in some cases to be neglected completely. KF smoothes out the effects of noise in the state variables estimated, by incorporating more information from reliable data than unreliable data. If mechanization was run in an unaided fashion, the errors in its output are passed from one iteration to another thus leading for the navigation states errors to drift with time. Therefore, error models are required for the analysis and estimation of different error sources associated with the RISS system. Since the errors in dynamic systems are variable in time, they are described by differential equations. Linearization of a non-linear dynamic system is the most common approach to derive a set of linear differential equations that defines the error states of a dynamic system. Figure 2 shows where the KF fits within the navigation solution. One of the main advantages of the KF, computation-wise, is that it is very convenient for real-time processing.

KF addresses the general problem of trying to estimate the state of a discrete-time process that is governed by the models:

1. State equation (System model or Process model):
  (4) $$x_{k+1}=F_{k+1,k}.x_{k}+G_{k}w_{k}$$
  where:
  **x** = Error state vector
  *F* = State transition matrix
  *G* = Noise coupling matrix
  **w** = System/Process noise.
2. Observation equation (Measurement model):
  (5) $$z_{k+1}=H_{k+1}.x_{k+1}+\nu_{k+1}$$
  where:
  **z** = Observation vector (*i.e.*, Measurement vector)
  *H* = Measurement Design matrix
  ***ν*** = Measurement noise.

The corresponding error-state system model used by KF is presented in Equation (6) where *δ***x**_k+1_ is the error-state vector, *a_forward_* is the acceleration derived from odometer data, *T_s_* is the sampling time, *w_k_* is a unit variance white Gaussian noise. The error-state vector has 7 error states. These are the latitude and longitude errors (*δϕ*, *δλ*), the East and North velocities errors (*δV_East_*, *δV_Noth_*), the azimuth error (*δA*), the stochastic gyroscope error (*δω_Z_*) and the residual errors associated with the odometer-derived acceleration (*δa_forward_*). The stochastic errors associated with the gyroscope and the odometer-derived acceleration are modeled by Gauss-Markov models, *γ_a_forward__* is the inverse of the autocorrelation time for odometer-derived acceleration noise, $\sigma_{a_{\mathrm{forward}}}^{2}$ is the variance of odometer-derived acceleration noise, *β_ω_z__* is the inverse of the autocorrelation time for gyroscope noise, $\sigma_{\omega_{z}}^{2}$ is the variance of gyroscope noise.

(6) $$\delta x_{k}=\underset{k+1}{\underset{︸}{\left[\begin{array}{c} \delta \varphi \\ \delta \lambda \\ {\delta V}_{\mathrm{East}}\\ {\delta V}_{\mathrm{North}}\\ \delta A\\ \delta a_{\mathrm{forward}}\\ {\delta \omega }_{z} \end{array}\right]}}=\left[I_{7\times 7}+\left(\begin{array}{ccccccc} 0 & 0 & 0 & \frac{1}{R_{M}+h} & 0 & 0 & 0\\ 0 & 0 & \frac{1}{(R_{N}+h)\;\text{cos}\;\varphi } & 0 & 0 & 0 & 0\\ 0 & 0 & 0 & 0 & a_{\mathrm{forward}}\;\text{cos}\;A & \text{sin}\;A & 0\\ 0 & 0 & 0 & 0 & -a_{\mathrm{forward}}\;\text{sin}\;A & \text{cos}\;A & 0\\ 0 & 0 & 0 & 0 & 0 & 0 & 1\\ 0 & 0 & 0 & 0 & 0 & -\gamma_{a_{\mathrm{forward}}} & 0\\ 0 & 0 & 0 & 0 & 0 & 0 & -\beta_{\omega_{z}} \end{array}\right)T_{s}\right]\;\underset{k}{\underset{︸}{\left[\begin{array}{c} \delta \varphi \\ \delta \lambda \\ {\delta V}_{\mathrm{East}}\\ {\delta V}_{\mathrm{North}}\\ \delta A\\ {\delta a}_{\mathrm{forward}}\\ {\delta \omega }_{z} \end{array}\right]}}+\left[\begin{array}{c} 0\\ 0\\ 0\\ 0\\ 0\\ \sqrt{{2\gamma }_{a_{\mathrm{forward}}}\sigma_{a_{\mathrm{forward}}}^{2}}\\ \sqrt{{2\beta }_{\omega_{z}}\sigma_{\omega_{z}}^{2}} \end{array}\right]w_{k}$$

Since loosely coupled integration is used, GPS position and velocity updates are used during the update stage of KF. The measurement model used by KF is:

(7) $${\delta z}_{k}={\left[\begin{array}{ccc} \varphi^{\mathrm{RISS}} & - & \varphi^{\mathrm{GPS}}\\ \lambda^{\mathrm{RISS}} & - & \lambda^{\mathrm{GPS}}\\ V_{\mathrm{East}}^{\mathrm{RISS}} & - & V_{\mathrm{East}}^{\mathrm{GPS}}\\ V_{\mathrm{North}}^{\mathrm{RISS}} & - & V_{\mathrm{North}}^{\mathrm{GPS}} \end{array}\right]}_{k+1}=\left[\begin{array}{ccccccc} 1 & 0 & 0 & 0 & 0 & 0 & 0\\ 0 & 1 & 0 & 0 & 0 & 0 & 0\\ 0 & 0 & 1 & 0 & 0 & 0 & 0\\ 0 & 0 & 0 & 1 & 0 & 0 & 0 \end{array}\right]\;{\left[\begin{array}{c} \delta \varphi \\ \delta \lambda \\ {\delta V}_{\mathrm{East}}\\ {\delta V}_{\mathrm{North}}\\ \delta A\\ \delta a_{\mathrm{forward}}\\ {\delta \omega }_{z} \end{array}\right]}_{k+1}+{\delta \nu }_{k+1}$$

## 3. Embedded System Design

**Figure 3.**
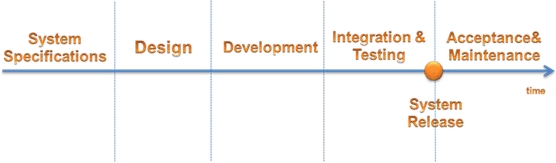
Embedded System Design Life Cycle.

Due to the complexity of the embedded systems, design methodologies are needed to guide the decisions of the designers when developing large systems. The selected design process, shown in Figure 3, also defines the constraints between different teams that are working on the system. The design methodology acts as a framework that will direct the work flow and organize the tasks in relation to the different phases in the system life cycle. It has to be noted that, unlike the waterfall model in which the flow of information and work from higher levels of abstraction (*i.e.*, earlier phases) to more detailed steps (*i.e.*, later phases) involves a limited amount of feedback to the upper higher levels of abstraction, the work focus can move from one phase to another in the backward direction allowing system refinement and optimization according to the experiences gained through the work [9,10].

### 3.1. System Requirements and Specifications Definition Phase

**Figure 4.**
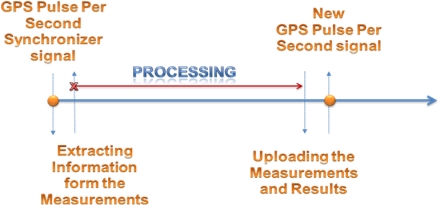
Measurements Synchronization, Information Extraction and Processing at 1 Hz rate.

The process of designing an embedded system starts with the definition of the system—the goal of the research work. The system definition describes what the embedded system is to be and do. The functional requirements for the real-time navigation embedded system are as follows:

1. Interface with the NovAtel SPAN unit [24] which includes the OEM4 GPS receiver [25], Crossbow inertial measurement unit (IMU) IMU300CC-100 (the Crossbow IMU is a MEMS grade low-end device that uses three orthogonal accelerometers and gyroscopes. However, the 2D RISS/GPS algorithm uses the vertical gyroscope only within the IMU which is aligned with the vehicle’s z-axis) [26] and a mobile robot’s encoders or the vehicle’s odometer via the ElmScan vehicle speed scan tool [27], using the universal asynchronous receiver transmitter (UART) serial communication interface. For each sensor, a UART channel is required.
2. Synchronize the data from the three sensors using the pulse per second (PPS) signal from the GPS, for a more reliable fusion solution.
3. Extract the information required from these sensors to get the position, velocity and heading from the GPS receiver; the angular rotation rate from the vertical gyroscope within the IMU which is aligned with the vehicle’s z-axis; and the vehicle’s speed from the wheel encoders or the odometer.
4. Once the synchronized extracted information is available, the KF for 2D RISS/GPS navigation algorithm is applied, to yield a consistent navigation solution per second. The rate of the navigation solution is 1 Hz due to utilizing the robot’s wheel encoders or the vehicle’s odometer in the system, where the speed readings are acquired at 1 Hz relative to the PPS synchronization signal. The navigation solution has to be computed and available before the new incoming GPS PPS synchronizer signal and measurements from the three sensors are available as shown in Figure 4.
  It has to be noted that the navigation solution computed by the RISS/GPS algorithm is bounded by the rate of system sensors. The solution computed by the developed real-time system is at a rate of 1 Hz due to the speed reading which is acquired from the robot’s wheel encoders or the vehicle’s odometer at a 1 Hz rate with respect to the arrival of the PPS signal from the GPS receiver. This rate is not a limitation of the RISS/GPS algorithm or the real-time system implementation. To improve the bounded rate of the navigation solution which is bounded by the 1 Hz rate measurements generated from the robot’s wheel encoders and the vehicle’s odometer OBDII, high resolution wheel encoders can be used for both the robot and the land vehicle. High resolution encoders will enable the reading of the encoder data at a higher rate while still preserving the accuracy of the measurements which can generate higher rate measurements; lower resolution encoders will cause quantization errors if read at higher rate, furthermore the OBD-II does not provide high rates.
5. The system is required to communicate with a PC via the UART interface to upload the measurements and the computed navigation solution to retrieve, visualize, and analyze the data.

The two approaches that are adopted to test the implemented real-time system are:

1. Using the measurements that are uploaded to the host machine, the solution of the navigation system in real-time is compared to the offline solution computed using the main algorithm that is coded in C which makes use of the uploaded measurements. If the results from both solutions are comparable, then the developed system has succeeded in delivering a real-time navigation solution.
2. The reference solution, which is generated from the NovAtel SPAN unit that contains a GPS receiver and a high-end high-cost tactical grade Honeywell IMU, the HG1700, will be used as a general reference, for assessing the performance of the low-cost developed navigation solution and the offline C solution.

### 3.2. Design Phase

In the design phase, two questions are answered. The first is concerned with the platform to be chosen that will fulfill the specifications of the embedded systems acquired in the first phase. The designer has the option to choose a μC, a DSP, an FPGA, or a combination of these platforms for the application to be developed. Once the platform is chosen, the designer has to decide on the tools that will assist the developers in programming the chosen platform.

A processor-based system based on off-the-shelf (OTS) microprocessor solutions is the traditional approach that designers have used in creating embedded systems. These types of traditional processor-based systems are available with a wide range of features and peripherals from a multitude of vendors. The process of selecting an OTS processor that can meet specific cost and functional requirements is time-consuming. It needs all the requirements to be gathered and solidified before the project development begins, which is seldom the case. Also, some of the low-end OTS solutions lack a dedicated floating-point unit which can boost the system performance for math centric algorithms. Due to the mentioned reasons, another approach is sought, which can allow the designer to tailor a processor and a specific set of features and peripherals for the application to be implemented [28].

Based on the above reasons, Xilinx’s MicroBlaze [29] soft-core processor was selected to develop the mobile multi-sensor navigation system. Similar to the OTS processors, an FPGA-based soft-core processor like the MicroBlaze can be programmed using high-level languages such as C/C++. MicroBlaze also provides many advantages compared to OTS processor solutions such as [28,30].

1. Customization: The designer of the FPGA-based embedded processor system has the total flexibility to add any combination of peripherals and controllers. A unique set of peripherals can also be designed for specific applications, and the designer has the privilege to add as many peripherals to meet the system requirements, which cannot be done when using an OTS processor. Future system features that didn’t have reason to exist in the initial release can also be added due to the space that is provided by an FPGA.
2. Multi-Processor System: As more complex embedded systems can benefit from the existence of multiple processors which can execute the tasks in parallel, by using a soft-core processor accompanying tools, the system designer can easily create a multi-processor based SOC. The only restriction to the number of processors that can be added is the availability of FPGA resources.
3. Hardware Acceleration: Hardware and software concurrent development and co-existence on a single chip, is one of the compelling reasons for choosing a soft core processor. System designers need not to worry, if a segment of the algorithm was found to be a bottleneck, as a custom co-processor or a hardware circuit that utilizes the FPGA parallelism can be designed to eliminate such problem.
4. Obsolescence Mitigation: Supporting extended project life expectance and component obsolescence mitigation is a difficult issue. FPGA soft-processors are an excellent solution in this case since the HDL code for the soft-processor can be purchased, and ownership of the HDL code can fulfill the requirements for product lifespan extension.

The soft-core processor is not without disadvantages. The processor will not operate at the same speeds or have the same performance as a hard-core processor. Unlike an off-the-shelf processor, the hardware platform for the FPGA embedded processor must be designed, where the embedded designer becomes the hardware processor system designer when an FPGA solution is selected. Also, due to the integration of the hardware and software platform design, the design tools are more complex, and the learning curve is steeper. The increased tool complexity and design methodology requires more attention from the embedded designer [30].

**Figure 5.**
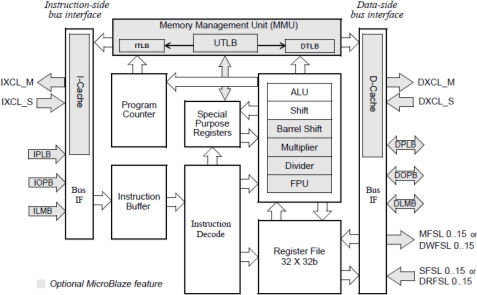
MicroBlaze Core Block Diagram [29].

The MicroBlaze is a 32-bit reduced instruction set computer (RISC) for use in FPGA designs targeting supported Xilinx Spartan or Virtex families of physical FPGA devices. Figure 5 shows a functional block diagram of the MicroBlaze core [29]. The MicroBlaze soft core processor is highly configurable, allowing the selection of a specific set of features for the system to be designed. Xilinx XPS software tool version 10.1.3 is used which corresponds to MicroBlaze version 7.10.d (For every Xilinx EDK version, there is only one corresponding MicroBlaze version which can be used. Newer MicroBlaze versions can be used only with a new Xilinx EDK release). The fixed feature set of the processor includes: (a) Thirty-two 32-bit general purpose registers (b) 32-bit instruction word with three operands and two addressing modes (c) 32-bit address bus and (d) Single issue pipeline. In addition to these fixed features, the MicroBlaze processor can be parameterized to allow selective enabling of additional functionality [29]. Some of the optional features are introduced in Table 1.

### 3.3. Development Phase

For developing an embedded system based on a soft-core processor, unlike the discrete processors where the hardware is already defined, the developer needs to start by customizing the soft-core processor to fit the application. Specific optional features and interfacing peripherals tailored for the application are added to the processor’s core.

Xilinx’s Embedded Development Kit (EDK) is a suite of tools and Intellectual Property (IP) that enables designing a complete embedded processor system for implementation in a Xilinx FPGA device. The EDK enables the design and integration of both the hardware and software using two main tools, Xilinx Platform Studio (XPS) and Software Development Kit (SDK).

**Figure 6.**
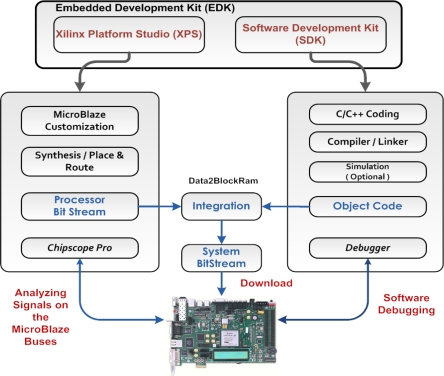
Hardware Customization and Software Development Flows Using Xilinx’s EDK.

The Xilinx Platform Studio is the development environment or graphical-user interface (GUI) used for designing the hardware portion of the embedded processor system. The SDK is an integrated development environment, complimentary to XPS, that is used for C/C++ embedded software application creation and verification. SDK is built on the Eclipse™ open-source framework, so this software development tool might appear familiar to software developers (Prior to EDK version 12.1 released in 2010, the designer had the option to use only XPS to develop both the hardware and software. Starting from 12.1, XPS is used for customizing the system hardware only, and the software has to be implemented on SDK.) [31]. Figure 6 illustrates the hardware design flow, starting from the hardware customization and resulting in the processor bit-stream, and the software design flow starting from C/C++ code development and resulting in the object code. The system bit-stream which contains both the processor bit-stream and the object code is then downloaded to the FPGA.

### 3.4. Hardware Customization

Virtex-4 ML402 evaluation kit powered by Xilinx Virtex-4 SX35 FPGA was utilized for building the real-time navigation system with the MicroBlaze processor in its core running at 100 MHz. The MicroBlaze was customized with four serial channel interfaces to communicate with the three sensors and the monitoring PC. In addition, general-purpose input-output (GPIO) channels were required for PPS and user interfacing, a timer for code profiling and an interrupt controller for the event-based system. The customized processor was also augmented with an IEEE-745 compliant single-precision floating point unit (FPU) provided via Xilinx cores in order to execute the navigation algorithm once the measurements are available in the least time possible. Although the FPU used is single-precision, the algorithm was developed making use of double-precision arithmetic which gives more accurate results. Consequently, software libraries were used by the compiler, provided with Xilinx SDK, to emulate the double-precision operations using the single-precision FPU, resulting in a slightly denser code but a far better navigation solution.

**Figure 7.**
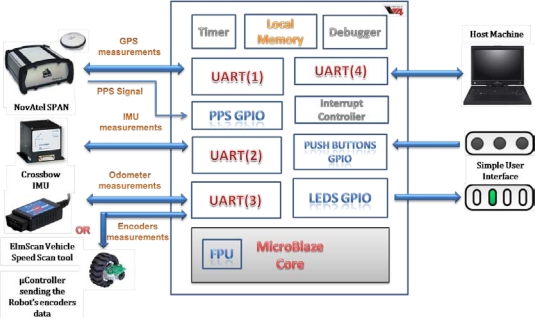
Customized MicroBlaze-based Navigation Embedded System.

Regarding the memory option that is used to store the instructions and data; the local memory [30], which utilizes Xilinx’s Block RAM (BRAM) memory blocks was selected and preferred over the choice to use the external SRAM or DDR memory combined with the cache option. Two memory cores of 128 KB each are used for storing the instructions and data. MicroBlaze accesses to BRAM take a single bus cycle. Since the processor and bus run at the same frequency in MicroBlaze, instructions stored in BRAM are executed at the full MicroBlaze processor frequency, thus the design achieves optimal memory performance as the code including the navigation algorithm was optimized to fit within the customized local memory. The customized MicroBlaze-based navigation system is shown in Figure 7.

### 3.5. Software Development

In designing the software, the bottom-up approach is used, where low abstraction modules are coded first, before moving to higher abstraction modules. We started by interfacing the sensors, and synchronizing the measurements according to the PPS signal, and then we moved to convert the MATLAB code to C and optimize the code for performance and code density. The lower level modules are tightly coupled with the system peripherals, while the higher level modules are generic modules that aren’t tied to specific hardware. Due to the use of interrupts, the system is event-triggered that is invoked when specific events occur such as acquiring one byte or one whole data packet from one of the UARTs that are used for interfacing the sensors, or the transition of the PPS signal from the low to high edge used for synchronization. Instead of using an operating system to control the hardware resources, the standalone Board Support Package (BSP), provided by Xilinx, is used to access the board/processor features directly without the use of a kernel or an operating system.

### 3.6. Measurement Synchronization

Time synchronization between GPS, IMU and odometer measurements is a common concern when implementing integrated systems. Since the NovAtel SPAN unit, the Crossbow IMU and the ElmScan 5 compact scan tools are three separate, self-contained subsystems, the clock difference and data transmission latency could cause data alignment discrepancies during the data fusion stage, using KF. Such alignment discrepancies may render the data fusion suboptimal [32].

**Figure 8.**
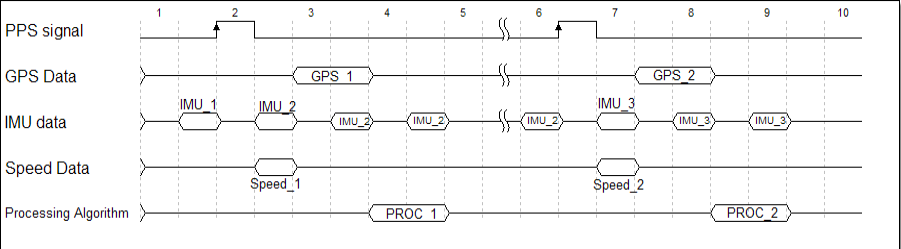
Timing Diagram showing the PPS Signal with respect to the GPS, IMU and speed data derived from either the odometer or the wheel encoders.

The GPS time is typically used as a time reference for multi-mobile sensor systems [32]. In addition to outputting positioning data and time messages through a serial data link, the NovAtel GPS receiver provide a one pulse-per-second electrical signal indicating the time of the turnover of each second. The alignment of the PPS signal edge to the standard GPS time is accurate to +/−50 ns [25]. The width of the PPS signal is 1 millisecond. Figure 8 shows the PPS signal with respect to the measurements from the three sensors.

For synchronizing the measurements, the PPS signal is connected to a GPIO core which is linked to an interrupt controller. The PPS signal is considered the system’s heartbeat and is tied to the highest interrupt priority within the interrupt controller. When an edge transition occurs on the line of the GPIO, from low-level to high-level, an interrupt occurs signaling the occurrence of a new second. The occurrence of the new second results in 2 operations, down-sampling all the yaw rate measurements from the z-axis gyroscope and acquiring a new speed measurement from the ElmScan tool or the robot’s wheel encoder processor. Shortly after the PPS signal is generated, the GPS data is received. When the GPS data is received, along with the down-sampled yaw rate measurement and the instant vehicle’s speed, the navigation algorithm processing can start. While the navigation algorithm is processing the synchronized measurements, the processor is forced to switch context to the IMU UART interrupt handlers in order to acquire the high-rate gyroscope measurements which occur at the rate of 200 Hz. Thus, no yaw rate measurements are lost.

### 3.7. Navigation Algorithm Conversion from MATLAB to C and Optimization

The conversion from MATLAB to C is done manually, instead of automatically using, for instance, the Embedded MATLAB approach. The embedded MATLAB approach, although is a faster way for algorithm conversion, needs the main MATLAB code to be altered in order to be compatible for the conversion process. It provides also less control over the generated C code, where the developer might need to optimize the code for a specific processor.

Although, the floating-point unit used in the MicroBlaze core is a single-precision, the algorithm was developed making use of the double-precision arithmetic. Consequently, the double-precision arithmetic operations are emulated using software libraries on the single-precision FPU, resulting in a slightly denser code but far better navigation solution. It has to be noted, that if the double-precision arithmetic was to be emulated on fixed-point hardware, the code would have been much denser.

After the navigation C code is implemented, the process of optimizing the code starts, that can result in faster code execution and less code density. For instance, instead of using the math *power*() function provided in C, to raise a specific variable to a power of 1.5, and using another *sqrt*() function, which is equivalent to raising a variable to the power of 0.5, the developer can choose the *power*() function to be used instead of the *sqrt*() function, Thus, extra memory that is needed to add the code space for both functions is eliminated resulting in a smaller code density.

### 3.8. Hardware Resources and Software Profiling

Table 2 presents the hardware resources that are utilized for building the whole navigation system. The logic cells used are 20% of the total available and the BRAM is 33.3% of the total available, which means that a lower-density FPGA can be used for implementing the system. Furthermore, if the same FPGA is used, then there is additional space to augment the system with more peripherals and coprocessors.

The code used on the MicroBlaze consumes 92% of the customized available memory and one iteration of the code takes a maximum of 35 milliseconds. The code profiling was done using the profiling timer which was initiated at the beginning of each iteration, and the number of clock cycles was then saved at the end of the iteration, to be sent to the host machine.

## 4. Experimental Results

### 4.1. Mobile Robot Testing Platform

**Figure 9.**
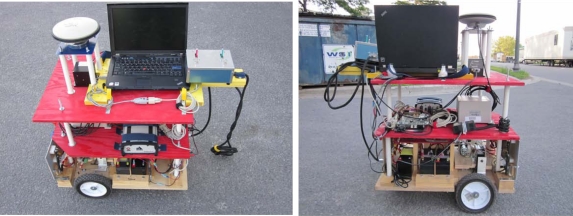
The Mobile Robot Testing Platform.

**Figure 10.**
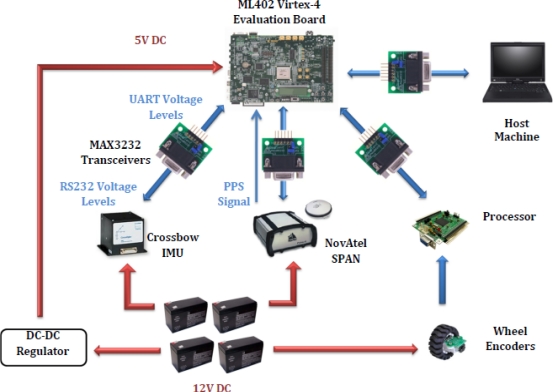
Mobile Robot Equipment Wiring Connections.

The real-time navigation system was implemented on Xilinx FPGA evaluation board ML402 featuring Virtex-4 SX35 and connected to the various sensors for acquiring the measurements. Figure 9 presents the experimental setup of the sensors and the FPGA evaluation kit on the robot. Figure 10 shows the wiring connections of the sensors mounted on the robot with the ML402 evaluation board. The power connections to the sensors and the evaluation board are also shown. It has to be mentioned that the host machine is not externally powered and depends on batteries which made the experiments on the mobile robot limited to a maximum of 1–1.5 hours.

A total of fifty trajectories were acquired using the mobile robot around the Royal Military College of Canada over 45 days, which were used in debugging the firmware on the navigation system. This number of trajectories would have been hard to acquire for testing and debugging the system using a land vehicle, which is more costly and requires more man power. Extensive trajectories were performed for both platforms, the mobile robot and the vehicle, and due to the results being consistent and similar, sample trajectories were selected that convey different information and trajectory challenges. 4.2. Mobile Robot—Trajectory (1)

**Figure 11.**
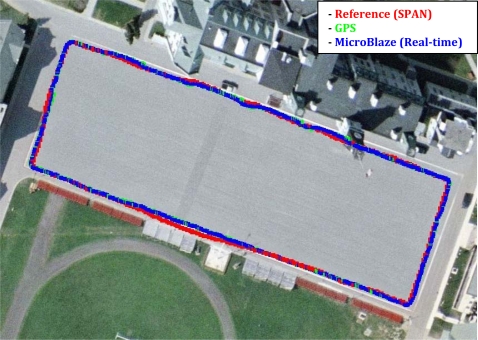
Mobile Robot Trajectory (1): Open Sky Area, with no Natural or Simulated GPS Outages.

**Figure 12.**
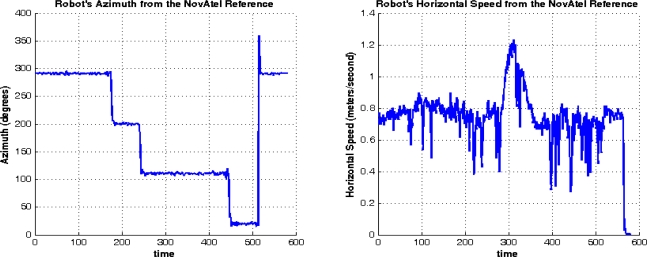
Mobile Robot Dynamics (Azimuth and Horizontal Speed) over Trajectory (1) from the Reference.

Trajectory (1) was acquired in an open sky area, where no natural GPS outages can be experienced and simulated GPS outages were not inserted. Figure 11 shows the trajectory that was computed by the developed real-time navigation system, and its comparison with the NovAtel SPAN reference solution and the GPS navigation solution. Figure 12 shows the robot’s dynamics represented by the azimuth and the horizontal speed from the NovAtel reference over the whole trajectory. The duration of trajectory (1) is 9.6 minutes.

**Figure 13.**
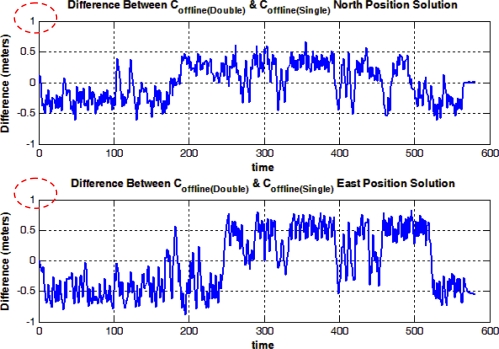
Mobile Robot Trajectory (1): Difference between C Double-Precision Offline and C Single-Precision Offline Solutions.

In Figure 13, the difference between the positioning solutions from the C double-precision offline algorithm (*i.e.*, The offline solution was computed using the main algorithm that is coded in C on the host machine which makes use of the uploaded measurements from the embedded system via the UART channel) and the C single-precision offline algorithm is presented, where the difference is in the range of 0.5 meter. The results show that the choice of emulating the double-precision on the MicroBlaze core is far more convenient in terms of accuracy, compared to using single-precision arithmetic which would have consumed less memory and less processing time.

**Figure 14.**
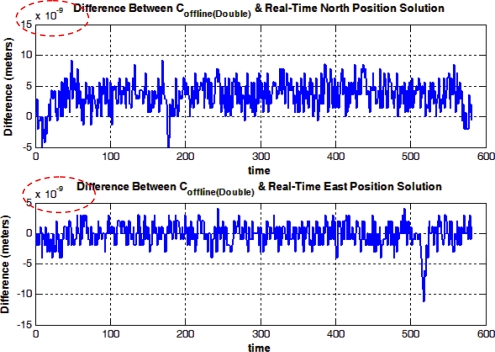
Mobile Robot Trajectory (1): Difference between C Double-Precision Offline and MicroBlaze Real-Time Solutions.

In Figure 14, the difference between the C double-precision offline solution evaluated on a PC and the MicroBlaze real-time solution with emulated double precision is presented, were the difference is in the magnitude of 10^−9^ meters. The difference can be due to the emulation of double-precision floating-point operations on the single-precision floating point unit present in the MicroBlaze core; however, the difference is negligible and it consequently shows that the real-time system succeeded in providing the same navigation solution as the one generated from the offline system.

**Figure 15.**
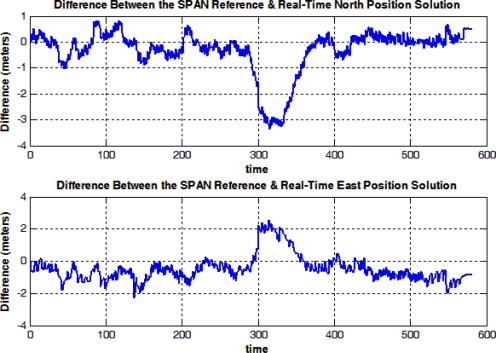
Mobile Robot Trajectory (1): Difference between NovAtel SPAN Reference and MicroBlaze Real Time Solutions.

Finally, the difference between the NovAtel SPAN reference solution and the real-time solution is shown in Figure 15. Both solutions are real-time, and the difference is due to the use of the low-cost MEMS-based IMU in the developed solution while the SPAN unit uses the high-end tactical grade HG1700 IMU. In general, the trajectory is open sky trajectory; however the part of the trajectory around 300 seconds from the start is when passing by a bleacher in the “Parade Square” area at the Royal Military College of Canada where some events take place. The GPS positioning solution was not the best at this point and it had about 3 to 4 meters of errors because of multipath, but still the standard deviation provided by the GPS receiver was good.

The off-the-shelf reference solution using a high-end tactical grade IMU, the HG1700, was able to provide the correct positioning solution despite the GPS with undetected multipath, because when using such high-end IMUs the balance of effect (*i.e.*, the balance of the covariance matrices) between the inertial and the GPS solutions inside the integrated solution is different than when using MEMS-based sensors. When using MEMS-based sensors, the integrated solution uses higher values in the covariance matrices because of the large errors present in these sensors which lead to relying more on GPS in the integrated when its standard deviation is good (*i.e.*, its covariance matrix will be good). So, the presented low-cost solution was slightly misled by GPS because of its good standard deviation. This fact can be seen when taking a closer look at Figure 11, in the middle of the bottom side of the rectangular trajectory, where the presented low-cost solution (blue) follows GPS (green) while the high-end reference solution (red) does not. It is to be noted that this scenario is still not a big problem in the presented low-cost solution because other scenarios with noticeably bad GPS position with misleading good standard deviation will not affect the integrated navigation solution because it will be detected and rejected by a GPS assessment routine that uses, in addition to the standard deviation, the motion constraints on land vehicles and speed readings (from wheel encoders or odometer) to detect and reject degraded GPS readings. However, since the GPS error in the current scenario was in the range of 3 to 4 meters and was not conflicting with the motion constraints it was still accepted and gained its way through to update the KF of the integrated solution.

The RMS and maximum errors in the east velocity, north velocity, North position and East position over trajectory (1) entirely are presented in Tables 3 and 4, respectively.

The first row of values in Tables 3 and 4 shows the RMS and maximum errors between the solution generated from both the C double-precision and C single-precision solutions. Again, the values reflect on the fact that the double-precision emulation was important to provide a more accurate navigation solution. This point is further confirmed by the 2nd row of values in Table 3, where it shows that the performance of the offline and real-time systems is essentially the same as the RMS error between solutions from both systems is in the order of 10^−9^ which is negligible. The 3rd row of values in Table 3 shows the RMS error from both the reference and real-time systems where the RMS error in the North and East positions is less than 1 meter.

### 4.3. Mobile Robot—Trajectory (2)

**Figure 16.**
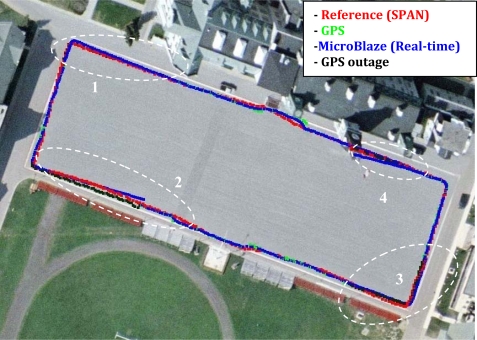
Mobile Robot Trajectory (2): Open Sky Area with Four Simulated GPS Outages.

Trajectory (2) is different from trajectory (1) as simulated outages were pre-programmed and inserted in the code at specific instants during the real-time processing. Simulated outages are used to imitate the effect of a GPS outage in areas where natural GPS outages aren’t experienced. Outages are introduced in the code to test how the navigation algorithm will perform in the absence of an update from the GPS, *i.e.*, in prediction mode based on the RISS only. The duration of all the simulated outages is 60 seconds. Trajectory (2) follows the same path as trajectory (1), and its duration is 9.3 minutes. Four outages were inserted at the seconds 120, 230, 400 and 525 respectively. Figure 16 shows trajectory (2) on an aerial map, from which the open sky area where the trajectory was acquired can be seen; the simulated outages locations are circled on the map.

Trajectory (2) was intended to be the same as trajectory (1) in order to show the effects of the intentionally simulated outages and the performance of the real-time system with respect to trajectory(1) which was analyzed before. Unlike trajectory (1), where the errors where analyzed over the whole trajectory; in trajectory (2), the performance of the real-time system was analyzed within the intentionally inserted outages. In Table 5, the RMS and the maximum horizontal position errors (*i.e.*, The horizontal position error is the square root of the square of the North and East position errors) during the four 60 second outages are presented, which indicate the performance of the real-time solution with respect to the NovAtel SPAN reference solution during the absence of the GPS update. The RMS error between the real-time and reference solutions reaches a maximum of 2.16 meters through outage (4). The performance of the position solution generated from the offline C double and single precisions is again analyzed for the four 60-second outages in trajectory (2) and is shown in Table 6. Again, the difference between both solutions is within a couple of meters indicating the importance of choosing the double-precision arithmetic for the RISS/GPS navigation algorithm instead of the single-precision. The difference between the position solution generated from the offline and the real-time systems is also analyzed within the four outages as shown in Table 6, where the RMS error is in the order of 10^−6^ for outage (2). The difference between the reference and the real-time systems’ solutions is then presented where it can be seen that the RMS error in the East and North positions over outage (4) is less than 2 meters to indicate that the performance of the real-time system which contains low-cost MEMS-based inertial sensors is very competitive with respect to the high-end, high-cost reference system.

### 4.4. Mobile Robot—Trajectory (3)

**Figure 17.**
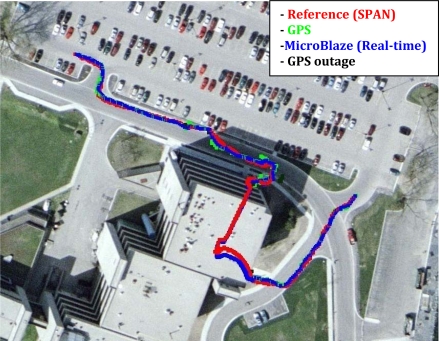
Mobile Robot Trajectory (3): Inside a building with a natural GPS Outage of 100 seconds duration.

**Figure 18.**
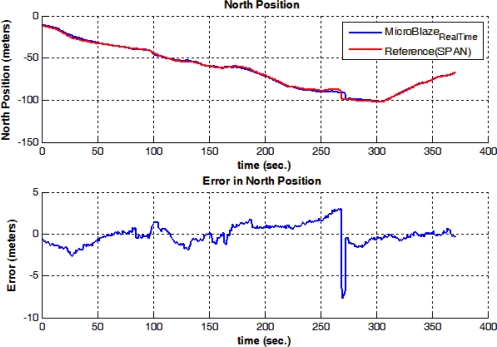
North Position Solution and Error in North Position between the Real-time and Reference Solutions during Trajectory (3).

**Figure 19.**
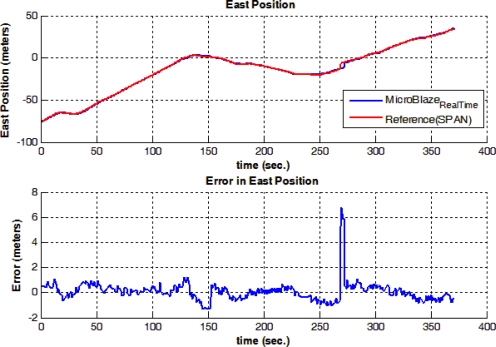
East Position Solution and Error in East Position between the Real-time and Reference Solutions during Trajectory (3).

Figure 17 shows trajectory (3), which is different from the previously presented trajectories, in having a portion of the trajectory acquired inside of a building. A natural GPS outage of 100 seconds occurred, which is equivalent to the time taken by the mobile robot from the entrance to the exit of the building. The natural outage was detected by the system which acted accordingly in providing a navigation solution in prediction only mode based on RISS readings; the solution still shows a very competitive performance with respect to the high-end, high-cost NovAtel SPAN reference system. The RMS error in the horizontal position during the outage is 2.39 meters, while the maximum error is 10.25 meters. Figures 18 and 19 show the error in North and East positions between the high-end reference solution and the developed low-cost real-time solution.

### 4.5. Land Vehicle Testing Platform

**Figure 20.**
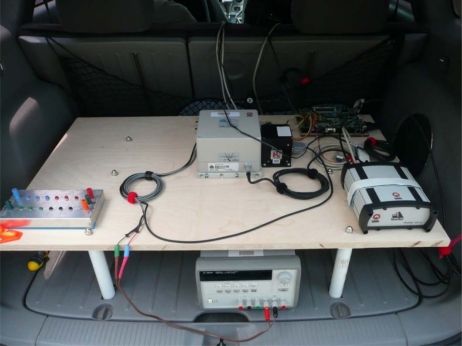
The Land Vehicle Experimental Setup.

After the firmware of the navigation system was debugged on the mobile robot and proven to give the same results as the C-code offline solution, the next step was to test the navigation system on a land vehicle where the wheel encoders on the mobile robot are replaced with the ElmScan vehicle speed scan tool that reads the vehicle speed readings through OBD-II interface. The experimental setup of the sensors and the FPGA evaluation kit on the land vehicle platform is shown in Figure 20.

**Figure 21.**
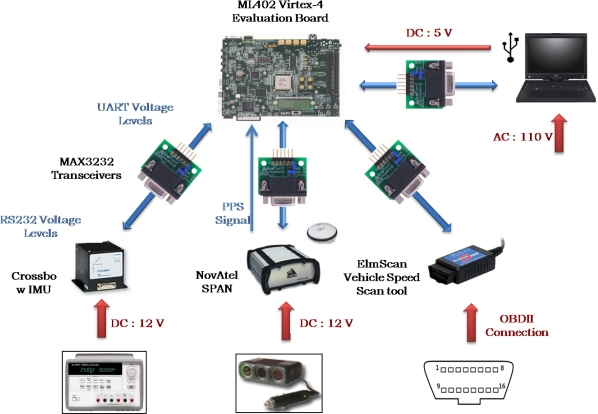
Land Vehicle Equipment Wiring Connections.

Figure 21 shows the wiring connections of the sensors with the ML402 evaluation board featuring Xilinx Virtex-4 FPGA on which the MicroBlaze soft-core processor is running. The power connections to the sensors and the evaluation board are also shown.

### 4.6. Land Vehicle—Trajectory (1)

**Figure 22.**
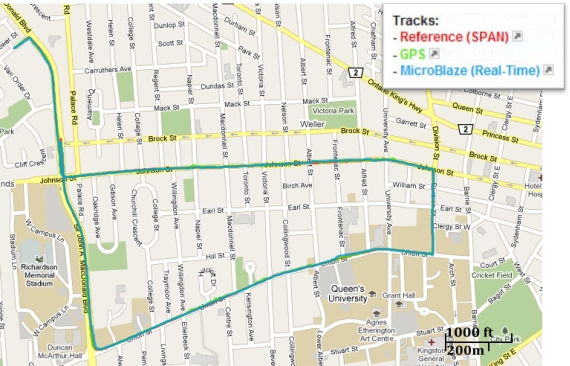
Land Vehicle Trajectory (1): Open Sky Area, with Minimum Natural Outages and no Simulated GPS Outages.

**Figure 23.**
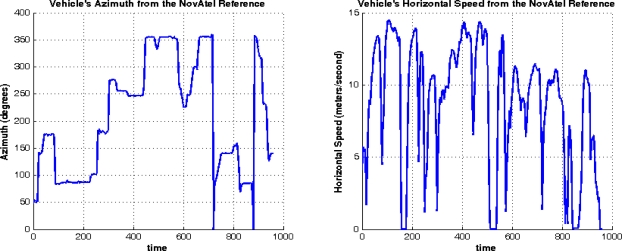
Land Vehicle Dynamics (Azimuth and Horizontal Speed) for Trajectory (1).

Trajectory (1) was acquired in an open sky area in Kingston around Queen’s University, where a small number of natural GPS outages can be experienced and simulated GPS outages were not inserted. Figure 22 shows the trajectory that was computed by the developed real-time navigation system, and its comparison with the NovAtel SPAN reference solution and the GPS navigation solution. Figure 23 shows the vehicle’s dynamics over the whole trajectory from the NovAtel reference solution. The duration of the trajectory is 9.2 minutes.

The differences between the offline, real-time and reference navigation solutions are presented in Tables 7 and 8 where the RMS and maximum errors in the East velocity, North velocity, East position, and North position over the whole trajectory are shown. The results for the land vehicle are comparable to the results for the mobile robot, from which it can be shown that the performance of the offline and real-time systems is very similar as the RMS error between the solutions generated from both systems is in the order of 10^−7^. It is to be noted that although the trajectory was taken in nearly open-sky, as mentioned previously a small number of natural GPS outages was experienced and GPS was also rejected in some areas mainly due to multipath caused by the trees on both sides of the street in these areas. This is the reason for the position error seen in Tables 7 and 8.

### 4.7. Land Vehicle—Trajectory (2)

**Figure 24.**
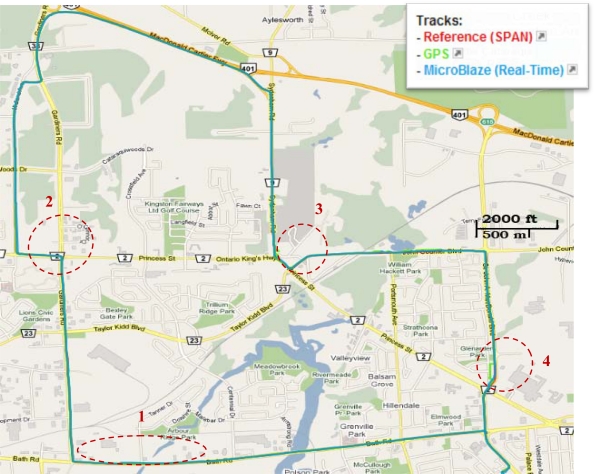
Land Vehicle Trajectory (2): Open Sky Area with Four Simulated GPS Outages.

**Figure 25.**
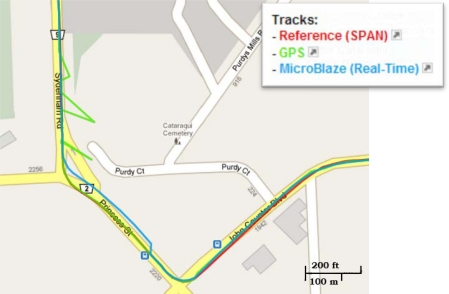
Land Vehicle Trajectory (2): Zooming-in outage (3).

**Figure 26.**
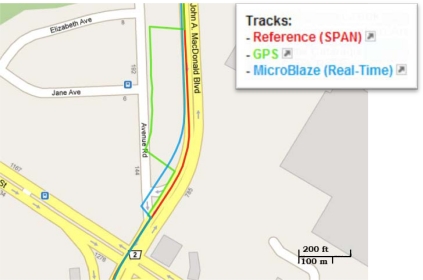
Land Vehicle Trajectory (2): Zooming-in outage (4).

Land vehicle Trajectory (2) is different from trajectory (1) as simulated outages were pre-programmed and introduced in the code at specific instants during the real-time processing. Four 60-second simulated outages are inserted in trajectory (2) at the seconds 300, 600, 1,000 and 1,300 respectively. The duration of the trajectory is 23.9 minutes. Figure 24 shows trajectory (2) in a map view, and the inserted GPS outages locations are encircled. Figure 25 shows a zoom-in view for simulated outage (3) in the trajectory; it can be seen that coincidently the GPS solution has some jumps outside the road, and of course the prediction-only real-time integrated solution was more consistent. This fact, however, clarify how the integrated solution, even when it runs in prediction-only mode is better than the GPS errors that might be caused by reflections without direct line of sight. Figure 26 shows a zoom-in view for outage (4) in the trajectory where again coincidently the error in the GPS solution was for a longer portion and larger in magnitude than outage (3); the developed system solution even in prediction-only mode is much more consistent. In Table 9, the RMS and the maximum horizontal position errors during the four 60-second outages are presented.

## 5. Conclusions

This paper discussed the process and challenges of realizing a mobile multi-sensor navigation algorithm such as the KF for 2D RISS/GPS integration algorithm. An embedded system design model was chosen to act as a framework for the work flow to be carried through the system life cycle starting from the system specification phase and ending with the system release. The realized system is capable of interfacing and communicating with a GPS receiver, a gyroscope and a vehicle’s odometer or a robot’s wheel encoders, synchronizing the sensors’ measurements with respect to the PPS signal and then applies the navigation algorithm yielding a reliable and accurate integrated navigation solution. Xilinx’s soft-core processor, MicroBlaze, on Virtex-4 FPGA was selected as the most suitable candidate for implementing the navigation system, where it provides the flexibility to choose or implement a set of features and peripherals that are tailored to the navigation system. The MicroBlaze also provides a single-precision floating point unit which was used to emulate the double-precision arithmetic embodied in the navigation algorithm, as the accuracy of the double-precision solution was higher than that of the single-precision solution. The error between the real-time emulated double-precision solutions when compared to the offline double-precision solution was in the range of 10^−9^ meters. The navigation system on the high-density Virtex-4 FPGA, utilized 20% of the total available logic cells 33.3% of the total available BRAM, which means that a lower-density FPGA can be used for implementing the system. The developed navigation system was tested first on a mobile robot to reveal system bugs and integration problems, and then on a land vehicle testing platform for further testing. The real-time solution from the implemented system when compared to the solution of a high-end navigation system, proved to be successful in providing a competitive consistent real-time navigation solution.

Using FPGA-based processors offer system designers the maximum flexibility to customize the processor to specific applications. The designers have even more flexibility to use multi-MicroBlaze cores for parallel computation. Co-designing software and hardware is also another option offered where designers can implement the bottleneck segments of the algorithm as a custom coprocessor or a hardware circuit that speeds up the algorithm. All these options promote the utilization of soft-core processors in a more attractive and feasible approach than the traditional OTS approach.

This paper doesn’t promote a soft-core processor from a specific vendor; however, it promotes using soft-core processors for implementing multi-sensor navigation systems as the future platform due to the offered advantages discussed in the paper. The paper demonstrated in length how embedded system design and development for FPGA-based processors is different than the traditional OTS processors, with an emphasis on navigation applications.

## Figures and Tables

**Table 1.** MicroBlaze Optional Features.

**Feature**	**MicroBlaze Version 7.2**
Processor pipeline depth	3/5
Hardware barrel shifter	option
Hardware divider	option
Hardware debug logic	option
Pattern Compare Instructions	option
Fast simplex link interfaces	Single-precision
Instruction and Data cache memory	option
Hardware exception support	option
Single-precision Floating Point Unit	option
Area or speed optimized	option
Floating-point conversion and square root instructions	option

**Table 2.** Hardware Resources and Memory Profiling.

**Resource**	**Used**	**Total Available**	**Utilization %**
Hardware Resources			
**Logic**	**5,935**	**30,720**	**19.3**
**Input/output**	**36**	**450**	**8.0**
**Block RAM**	**64**	**192**	**33.3**
**DSP**	**7**	**192**	**3.6**

Software Memory Profiling			
**Instruction Memory**	**117.3 KB**	**128 KB**	**91.6**

**Table 3.** RMS Error in the East Velocity, North Velocity, North and East Positions over Mobile Robot Trajectory (1).

**East Velocity (m/s)**	**North Velocity (m/s)**	**North Position (m)**	**East Position (m)**
RMS Error between the C Double-Precision and C Single-Precision Offline Solutions			
**4.12788 × 10^−2^**	**7.98887 × 10^−3^**	**2.96969 × 10^−1^**	**4.91848 × 10^−1^**
RMS Error between the C Double-Precision Offline and MicroBlaze Real-Time Solutions			
**7.83599 × 10^−10^**	**1.44185 × 10^−9^**	**4.16753 × 10^−9^**	**1.85211 × 10^−9^**
RMS Error between the NovAtel SPAN Reference and MicroBlaze Real-Time Solutions			
**9.60578 × 10**^−2^	**1.11987 × 10**^−1^	**9.05270 × 10**^−1^	**9.39925 × 10**^−1^

**Table 4.** Maximum Error in the East Velocity, North Velocity, North and East Positions over Mobile Robot Trajectory (1).

**East Velocity (m/s)**	**North Velocity (m/s)**	**North Position (m)**	**East Position (m)**
Maximum Error between the C Double-Precision and C Single-Precision Offline Solutions			
**7.49309 × 10^−2^**	**3.54033 × 10^−2^**	**6.65903 × 10^−1^**	**8.85448 × 10^−1^**
Maximum Error between the C Double-Precision Offline and MicroBlaze Real-Time Solutions			
**1.15944 × 10^−8^**	**2.82925 × 10^−8^**	**9.18873 × 10^−9^**	**1.11810 × 10^−8^**
Maximum Error between the NovAtel SPAN Reference and MicroBlaze Real-Time Solutions			
**4.06407 × 10^−1^**	**4.43351 × 10^−1^**	**3.34354**	**2.56632**

**Table 5.** RMS and Maximum Horizontal Position Error for the Four 60 seconds Outages Introduced in Mobile Robot Trajectory (2).

**Outage (1)**	**Outage (2)**	**Outage (3)**	**Outage (4)**
RMS Horizontal Position Error between the SPAN Reference and MicroBlaze Real-Time Solutions (m)			
**9.38501 × 10^−1^**	**1.40681**	**1.06658**	**2.16077**
Maximum Horizontal Position Error between the SPAN Reference and MicroBlaze Real-Time Solutions (m)			
**1.65584**	**2.48860**	**1.79489**	**3.08822**

**Table 6.** RMS Error in the Latitude and Longitude Solutions over the Four Outages introduced in Mobile Robot Trajectory (2).

	**North position (m)**	**East position (m)**
**Outage (1)**	RMS Error between the C Double-Precision and C Single-Precision Offline Solutions	
	**3.00674**	**6.42866**
	RMS Error between the C Double-Precision Offline and MicroBlaze Real-Time Solutions	
	**1.37906 × 10^−8^**	**4.92348 × 10^−8^**
	RMS Error between the NovAtel SPAN Reference and MicroBlaze Real-Time Solutions	
	**7.91314 × 10^−1^**	**5.04586 × 10^−1^**
**Outage (2)**	RMS Error between the C Double-Precision and C Single-Precision Offline Solutions	
	**3.39406**	**4.07745**
	RMS Error between the C Double-Precision Offline and MicroBlaze Real-Time Solutions	
	**1.38649 × 10^−6^**	**4.47223 × 10^−7^**
	RMS Error between the NovAtel SPAN Reference and MicroBlaze Real-Time Solutions	
	**4.42573 × 10^−1^**	**1.33538**
**Outage (3)**	RMS Error between the C Double-Precision and C Single-Precision Offline Solutions	
	**1.45689**	**3.67700**
	RMS Error between the C Double-Precision Offline and MicroBlaze Real-Time Solutions	
	**3.14829 × 10^−7^**	**2.40989 × 10^−7^**
	RMS Error between the NovAtel SPAN Reference and MicroBlaze Real-Time Solutions	
	**7.32207 × 10^−1^**	**7.75539 × 10^−1^**
**Outage (4)**	RMS Error between the C Double-Precision and C Single-Precision Offline Solutions	
	**8.03891 × 10^−1^**	**3.30091**
	RMS Error between the C Double-Precision Offline and MicroBlaze Real-Time Solutions	
	**6.60678 × 10^−9^**	**2.16253 × 10^−9^**
	RMS Error between the NovAtel SPAN Reference and MicroBlaze Real-Time Solutions	
	**1.84932**	**1.11755**

**Table 7.** RMS Error in the East Velocity, North Velocity, North and East Positions over Land Vehicle Trajectory (1).

**East Velocity (m/s)**	**North Velocity (m/s)**	**North Position (m)**	**East Position (m)**
RMS Error between the C Double-Precision and C Single-Precision Offline Solutions			
**1.78523 × 10^−1^**	**9.60298 × 10^−3^**	**3.76213 × 10^−1^**	**4.98008 × 10^−1^**
RMS Error between the C Double-Precision Offline and MicroBlaze Real-Time Solutions			
**5.85936 × 10^−8^**	**1.48418 × 10^−8^**	**1.19570 × 10^−7^**	**3.67245 × 10^−7^**
RMS Error between the NovAtel SPAN Reference and MicroBlaze Real-Time Solutions			
**6.73738 × 10^−1^**	**5.757354 × 10^−1^**	**6.42297**	**7.79358**

**Table 8.** Maximum Error in the East Velocity, North Velocity, North and East Positions over Land Vehicle Trajectory (1).

**East Velocity (m/s)**	**North Velocity (m/s)**	**North Position (m)**	**East Position (m)**
Maximum Error between the C Double-Precision and C Single-Precision Offline Solutions			
**1.405039 × 10^−1^**	**6.68926 × 10^−2^**	**1.24497**	**2.82222**
Maximum Error between the C Double-Precision Offline and MicroBlaze Real-Time Solutions			
**4.55655 × 10^−7^**	**1.47957 × 10^−7^**	**1.02348 × 10^−6^**	**3.84857 × 10^−6^**
Maximum Error between the NovAtel SPAN Reference and MicroBlaze Real-Time Solutions			
**4.50865**	**3.02078**	**2.01342 × 10**	**3.63886 × 10**

**Table 9.** RMS and Maximum Horizontal Position Error for the Four 60 seconds Outages Introduced in Land Vehicle Trajectory (2).

**Outage (1)**	**Outage (2)**	**Outage (3)**	**Outage (4)**
RMS Horizontal Position Error between the SPAN Reference and MicroBlaze Real-Time Solutions (m)			
**8.91707**	**1.40813 × 10**	**1.12782 × 10**	**1.63253 × 10**
Maximum Horizontal Position Error between the SPAN Reference and MicroBlaze Real-Time Solutions (m)			
**1.66452 × 10**	**2.37866 × 10**	**1.57619 × 10**	**2.39850 × 10**
